# Errors generated by a point-of-care CD4+ T-lymphocyte analyser: a retrospective observational study in nine countries

**DOI:** 10.2471/BLT.14.146480

**Published:** 2015-06-25

**Authors:** Emmanuel Fajardo, Carol Metcalf, Erwan Piriou, Monique Gueguen, David Maman, Pascale Chaillet, Vivian Cox, Maryam B Rumaney, Syanness Tunggal, Cara Kosack, Teri Roberts

**Affiliations:** a Médecins Sans Frontières (MSF), Southern Africa Medical Unit (SAMU), Waverly Business Park, 303 A&B, Cape Town, South Africa.; b MSF, Operational Centre Amsterdam, Netherlands.; c MSF, Operational Centre Paris, France.; d MSF, Epicentre Paris, France.; e MSF, Operational Centre Brussels, Belgium.; f MSF, Khayelitsha, South Africa.; g MSF, Roma, Lesotho.; h MSF, Diagnostic Network, Amsterdam, Netherlands.; i MSF, Access Campaign, Geneva, Switzerland.

## Abstract

**Objective:**

To estimate the proportion of invalid results generated by a CD4+ T-lymphocyte analyser used by Médecins Sans Frontières (MSF) in field projects and identify factors associated with invalid results.

**Methods:**

We collated 25 616 CD4+ T-lymphocyte test results from 39 sites in nine countries for the years 2011 to 2013. Information about the setting, user, training, sampling technique and device repair history were obtained by questionnaire. The analyser performs a series of checks to ensure that all steps of the analysis are completed successfully; if not, an invalid result is reported. We calculated the proportion of invalid results by device and by operator. Regression analyses were used to investigate factors associated with invalid results.

**Findings:**

There were 3354 invalid test results (13.1%) across 39 sites, for 58 Alere Pima^TM^ devices and 180 operators. The median proportion of errors per device and operator was 12.7% (interquartile range, IQR: 10.3–19.9) and 12.1% (IQR: 7.1–19.2), respectively. The proportion of invalid results varied widely by country, setting, user and device. Errors were not associated with settings, user experience or the number of users per device. Tests performed on capillary blood samples were significantly less likely to generate errors compared to venous whole blood.

**Conclusion:**

The Alere Pima CD4+ analyser generated a high proportion of invalid test results, across different countries, settings and users. Most error codes could be attributed to the operator, but the exact causes proved difficult to identify. Invalid results need to be factored into the implementation and operational costs of routine CD4+ T-lymphocyte testing.

## Résumé

**Objectif:**

Estimer la proportion de résultats non valides générés par l'analyseur de lymphocytes T CD4+ utilisé par Médecins Sans Frontières (MSF) dans les projets de terrain et déterminer les facteurs associés aux résultats non valides.

**Méthodes:**

Nous avons rassemblé 25 616 résultats de tests des lymphocytes T CD4+ réalisés sur 39 sites dans neuf pays entre 2011 et 2013. Un questionnaire a permis d'obtenir des informations sur le lieu, l'utilisateur, la formation, la méthode d'échantillonnage et les réparations effectuées sur l'appareil. L'analyseur réalise une série de contrôles afin de garantir que toutes les étapes de l'analyse se déroulent correctement; si ce n'est pas le cas, il indique un résultat non valide. Nous avons calculé la proportion de résultats non valides par appareil et par opérateur. Des analyses de régression ont permis de rechercher les facteurs associés aux résultats non valides.

**Résultats:**

Nous avons relevé 3354 résultats de tests non valides (13,1%) sur les 39 sites, avec 58 appareils Alere PimaTM et 180 opérateurs. La proportion moyenne d'erreurs par appareil et par opérateur était respectivement de 12,7% (intervalle interquartile, IQR: 10,3–19,9) et de 12,1% (IQR: 7,1-19,2). La proportion de résultats non valides variait considérablement en fonction du pays, du lieu, de l'utilisateur et de l'appareil. Les erreurs n'étaient pas associées aux lieux, à l'expérience de l'utilisateur ou au nombre d'utilisateurs par appareil. Les tests effectués sur des prélèvements de sang capillaire étaient nettement moins susceptibles de générer des erreurs que ceux réalisés sur du sang veineux total.

**Conclusion:**

L'analyseur de CD4+ Alere Pima a généré une proportion élevée de résultats de tests non valides, dans différents pays, différents lieux et avec différents utilisateurs. La plupart des codes d'erreur ont pu être imputés à l'opérateur, mais les causes exactes se sont avérées difficiles à déterminer. Les résultats non valides doivent être pris en compte dans les coûts de mise en œuvre et d'exploitation des tests de routine des lymphocytes T CD4+.

## Resumen

**Objetivo:**

Estimar la proporción de resultados no válidos generados por un analizador de linfocitos CD4+ T utilizado por Médicos sin Fronteras (MSF) en proyectos de campo e identificar los factores asociados a los resultados no válidos.

**Métodos:**

Se recopilaron 25 616 resultados de pruebas de linfocitos CD4+ T de 39 localizaciones en nueve países entre los años 2011 y 2013. La información sobre el entorno, los usuarios, la formación, la técnica de muestreo y el historial de reparación del dispositivo se obtuvo a través de un cuestionario. El analizador lleva a cabo una serie de chequeos para asegurar que se completan con éxito todos los pasos del análisis; si no es así, se informa de un resultado no válido. Se calculó la proporción de resultados no válidos por dispositivo y operador. Se utilizaron análisis de regresión para investigar los factores asociados con los resultados no válidos.

**Resultados:**

Hubo 3354 resultados no válidos de pruebas (13,1%) en 39 localizaciones, para 58 dispositivos Alere Pima^TM^ y 180 operadores. La proporción media de errores por dispositivo y operador fue del 12,7% (rango intercuartílico, RIC: 10,3–19,9) y del 12,1% (RIC: 7,1-19,2), respectivamente. La proporción de resultados no válidos varió ampliamente entre países, entornos, usuarios y dispositivos. Los errores no estaban asociados con los entornos, la experiencia del usuario o el número de usuarios por dispositivo. Las pruebas realizadas con muestras de sangre capilar tenían menos posibilidades de generar error que las realizadas con sangre entera venosa.

**Conclusión:**

El analizador Alere Pima CD4+ generó una alta proporción de resultados no válidos de pruebas en diferentes países, entornos y usuarios. La mayoría de los códigos de error podrían atribuirse al operador, pero resultó difícil identificar las causas exactas. Los resultados no válidos de las pruebas rutinarias de linfocitos CD4+ T deberían tenerse en cuenta en la aplicación y los costes operacionales.

## ملخص

**الغرض:**

تقدير نسبة النتائج غير الصالحة الصادرة عن جهاز تحليل خلايا اللمفاويات التائية ‎CD4+ الذي تستخدمه منظمة أطباء بلا حدود (MSF) في المشروعات الميدانية وتحديد العوامل المرتبطة بالنتائج غير الصالحة.

**الطريقة:**

لقد جمعنا 25,616 نتيجة اختبار لخلايا اللمفاويات التائية CD4+ من 39 موقعًا في تسعة بلدان في الفترة ما بين عاميّ 2011 إلى 2013. وتم الحصول على معلومات عن البيئة، والمستخدمين، والتدريب، وتقنية سحب العينات، وتاريخ إصلاح الجهاز عن طريق الاستبيان. يقوم جهاز التحليل بإجراء سلسلة من عمليات التحقق للتأكد من اكتمال جميع خطوات التحليل بنجاح؛ وفي حال عدم نجاحها، فسيتم الإبلاغ عن صدور نتيجة غير صالحة. وقد احتسبنا نسبة النتائج غير الصالحة الصادرة عن الجهاز وعن المُشغِّل. تم استخدام تحاليل التحوف لاستقصاء العوامل المرتبطة بالنتائج غير الصالحة.

**النتائج:**

كانت هناك 4335 نتيجة اختبار غير صالحة (بنسبة تبلغ 13.1%) عبر 39 موقعًا لعدد 58 جهازًا من طراز Alere Pima ^TM ^‎ و180 من المُشغِّلين. بلغت نسبة متوسط الأخطاء لكل جهاز ومُشغِّل 12.7% (المدى الربيعي: 10.3–19.9) و12.1% (المدى الربيعي: 7.1–19.2)، على التوالي. واختلفت نسبة النتائج غير الصالحة بشكل واسع حسب البلد والبيئة والمستخدم والجهاز. لم تكن الأخطاء مرتبطة ببيئة الاستخدام، ولا خبرة المستخدم، ولا عدد المستخدمين في كل جهاز. وكان احتمال صدور الأخطاء في الاختبارات التي أجريت على عينات الدم الشعري واردًا بدرجة أقل كثيرًا بالمقارنة مع عينات الدم الكامل الوريدي.

**الاستنتاج:**

أصدر جهاز التحليل Alere Pima CD4+‎ نسبة عالية من نتائج الاختبار غير الصالحة، عبر بلدان وبيئات استخدام ومستخدمين مختلفين. يمكن عزو معظم رموز الخطأ إلى المُشغِّل، ولكن ثبت أنه من الصعب تحديد الأسباب الحقيقية. هناك حاجة لمراعاة النتائج غير الصالحة في تكاليف التنفيذ والتشغيل لاختبار خلايا اللمفاويات التائية CD4+‎ الروتيني.

## 摘要

**目的:**

旨在评估由无国界医生组织  (MSF)  用于实地项目的 CD4+ T-淋巴细分析器产生无效结果的比例，并确认与无效结果相关的因素。

**方法:**

我们从 9 个国家的 39 个医疗点中获取了 25 616 项在 2011 年至 2013 年期间关于 CD4+ T-淋巴细胞的测试结果。通过调查问卷获得了关于环境、用户、培训、采样技术和设备维修记录的信息。 分析器会进行一系列检查，以确保分析的所有步骤都能成功完成；如果不能，则报告无效结果。 我们通过设备和操作员计算了无效结果的比例。 回归分析用于调查与无效结果有关的因素。

**结果:**

在 34 个医疗点的 58 个 Alere Pima^TM^ 设备和 180 名操作员中，出现了 3354 项无效测试结果  (13.1%)。 每个设备和操作员的错误比例中值分别为 12.7%（四分位差，IQR： 10.3–19.9)  和 12.1% (IQR: 7.1–19.2)。 无效结果的比例因国家、环境、用户和设备而异。 每个设备的错误与环境、用户经验或用户数量无关。 与静脉全血相比，关于毛细管血样的测试出现错误的可行性更低。

**结论:**

Alere Pima CD4+ 分析器在不同国家、环境和用户中出现无效测试结果的比例很高。 大多数错误代码归因于操作员，但是很难确认出确切的原因。 无效结果需在 CD4+ T-淋巴细胞常规测试的实施和操作成本中作为因素纳入考虑。

## Резюме

**Цель:**

Оценить долю недействительных результатов, выдаваемых анализатором количества CD4+ T-лимфоцитов в полевых условиях в рамках проектов организации «Врачи без границ» (Médecins Sans Frontières, MSF), и выявить факторы, вызывающие недействительные результаты.

**Методы:**

Мы сравнили 25 616 результатов определения CD4+ T-лимфоцитов в 39 медицинских учреждениях из девяти стран за два года (с 2011 по 2013 год). Информация относительно условий проведения анализа, а также сведения о пользователе, его обучении, методике отбора проб и истории ремонта измерительного прибора была получена в ходе анкетирования. Анализатор выполняет ряд проверок для того, чтобы удостовериться в успешном выполнении всех этапов анализа. В противном случае выдается сообщение о недействительном результате. Мы рассчитали долю недействительных результатов для каждого устройства и каждого оператора. Для изучения факторов, вызывающих недействительные результаты, был проведен регрессионный анализ.

**Результаты:**

В 39 учреждениях, где использовалось 58 устройств серии Alere Pima^TM^ и работало 180 операторов, было выявлено 3354 недействительных результата исследований (13,1%). Медианная доля ошибок в пересчете на устройство и оператора составила 12,7% (межквартильный размах, МКР: 10,3–19,9) и 12,1% (МКР: 7,1–19,2) соответственно. Доля недействительных результатов в значительной мере варьировалась в зависимости от страны, условий проведения анализа, пользователя и устройства. Ошибки не были связаны с условиями проведения анализа, опытом пользователя или количеством пользователей для одного и того же устройства. Если для анализа использовалась кровь из пальца, появление недействительных анализов было значительно менее вероятно, чем при использовании проб венозной крови.

**Вывод:**

Анализатор Alere Pima CD4+ выдает значительный процент недействительных результатов в зависимости от страны, условий проведения анализа и участия различных операторов. Возникновение большинства ошибок связано с работой операторов, но определить точную причину оказалось затруднительно. Недействительные результаты следует учитывать при определении расходов на внедрение и осуществление регулярных подсчетов количества CD4+ T-лимфоцитов.

## Introduction

The CD4+ T-lymphocyte count is the method recommended by the World Health Organization (WHO) to assess eligibility for antiretroviral treatment (ART).^1^^,^^2^ The CD4+ count also guides the clinical management of people living with human immunodeficiency virus (HIV).^2^ WHO recommends testing of viral load to detect treatment failure, but the CD4+ count continues to be used for ART monitoring if viral load cannot be tested.^3^ In settings where viral load can be determined, WHO suggests that routine CD4+ monitoring may be reduced or stopped for adults who are virologically stable.^4^^,^^5^

Increased availability of ART has driven the establishment of laboratories analysing CD4+ count, but access to CD4+ testing requires adequate laboratory capacity and the means to transport specimens.^6^^,^^7^ Point-of-care CD4+ testing provides rapid results without the need to transport specimens and is considered an important tool to improve patient retention in care before treatment initiation.^8^^–^^11^

The Alere Pima CD4+ analyser (Alere Technologies, Jena, Germany) was commercially launched in 2010. It is an automated analyser intended for counting CD4+ cells in capillary or venous whole blood within 20 minutes. The analyser is portable; can be operated with a rechargeable battery; contains dried thermostable reagents and can be stored at room temperature. The analyser performs a series of checks to ensure that all steps of the analysis are completed successfully, if not, an invalid result is reported. It is recommended that internal Pima Bead cartridges are analysed daily for quality control purposes. The analyser fulfils most WHO criteria for point-of-care tests^12^ and is on the WHO list of prequalified diagnostics.^13^

The analyser has been extensively evaluated with favourable diagnostic performance at laboratory level^14^^–^^25^ and with acceptable results at clinic level,^26^^–^^40^ in mobile clinics^41^ and in communities.^42^^,^^43^ Some studies showed higher variability of results at clinic level with capillary sampling.^44^^–^^46^ A recent meta-analysis showed that the analyser was comparable in performance to laboratory-based methods. The analyser identifies patients for treatment at the 350 CD4+ cells/μL threshold with a sensitivity of 91.6% and specificity of 94.8%.^47^

In 2011, Médecins Sans Frontières (MSF) introduced the analyser in various projects to make CD4+ counting more accessible and to reduce loss to follow-up. During the implementation phase, several MSF sites reported a relatively high proportion of errors. Previous studies have reported invalid results ranging from 2% to 15%.^29^^,^^32^^,^^38^^,^^39^ However, these studies have been conducted under validation conditions and there is a lack of data describing errors under routine field conditions. Given the relatively high proportion of invalid results reported at MSF sites, we carried out a retrospective analysis of analyser data across a variety of settings. The objective of the study was to describe the proportion of CD4+ test errors and identify factors associated with these errors. We hypothesized that invalid results might be related to the experience of the operators or to the type of blood sample used.

## Methods

### Study population

We conducted a retrospective, observational, cross-sectional study using routine data from 39 MSF-supported sites using the analyser in the Central African Republic, the Democratic Republic of the Congo, Guinea, India, Kenya, Lesotho, Malawi, Mozambique and South Africa, between 1 January 2011 and 30 June 2013. Analysers were introduced in laboratories, primary health-care clinics, mobile clinics and in communities. HIV-positive individuals requiring ART eligibility assessment, and in some instances, ART monitoring, had CD4+ counts done on capillary or venous blood samples. The study protocol was submitted to the MSF ethics review board and exempted from ethics review because it complied with the standards for routinely collected data analyses.

### Training

Laboratory technicians, clinicians and lay workers, were trained either by MSF-trained personnel or by the local Alere representative before use of the analyser. On-site training was conducted in half a day and focused on device operation, sampling technique, quality control and data management. In Lesotho and South Africa, a three-day centralized course in Alere’s training department in Cape Town was also provided to key staff who, in turn, trained multiple users on-site.

### Sampling technique

Venous blood samples were used at all sites in the Central African Republic, the Democratic Republic of the Congo, Guinea, India, Kenya, Malawi and two sites in South Africa; capillary sampling was used in Lesotho, Mozambique and the remaining nine sites in South Africa. All sites in the Central African Republic, the Democratic Republic of the Congo, Guinea, India, Kenya and Malawi used a fixed volume micropipette to transfer 25 µL of venous blood to the cartridge. Two sites in South Africa used a plain capillary tube for cartridge filling. Capillary sampling was performed using the Alere recommended safety lancet (Sarstedt, Nümbrecht, Germany) at remaining sites in South Africa and all sites in Mozambique and Lesotho.

### Quality control

Daily quality control was done at all sites using the manufacturer-supplied bead standards (with normal and low CD4+ cell counts) before testing patient samples. Eleven sites in South Africa were also enrolled in a regional proficiency testing programme and tested stabilized whole blood samples with normal or low CD4+ counts every two months.^48^ At most sites, dedicated paper-based registers were implemented to capture test results. None of the sites made use of the Alere online data portal during the study period. Test results and quality control data were analysed periodically in each country by a laboratory coordinator.

### Data collection

Archived computer files containing CD4+ test results and quality control data were collated from each device. Information about the setting, user, training, sampling technique and device repair history were obtained by questionnaire, completed by the laboratory coordinator in each country. To ensure a representative number of tests per device and to eliminate errors due to inexperienced operators, we excluded data from devices with less than 50 tests, users performing less than 50 tests or unknown operators.

### Analysis

We calculated the proportion of invalid CD4+ test results by device and by operator. Factors associated with invalid results were analysed using the binreg command in Stata version 13.0 (StataCorp. LP, College Station, United States of America). To determine the effects of user type and setting, we performed a series of analyses, restricted to those countries with comparable user type and setting.

We carried out the following comparisons: (i) tests done at clinics by lay workers versus tests done by clinicians in South Africa; (ii) tests done by laboratory technicians at clinics versus tests done at laboratories in the Central African Republic and the Democratic Republic of the Congo, and (iii) tests done by lay health workers in mobile settings versus tests done in clinics in Lesotho, Mozambique and South Africa. For each of these three comparisons, we calculated the frequency of errors stratified by country and tested for effect modification by country using the Mantel-Haenszel *χ^2^* test. If there was no effect modification by country, we used generalized linear regression models to assess associations between error frequency and user type or setting. The *χ*^2^ test for categorical variables and the two-sample test of proportions were used to assess the significance of differences; a *P*-value of less than 0.05 was considered statistically significant.

## Results

Between January 2011 and June 2013, there were 27 019 records in the data set. The exclusion criteria led to 1403 tests being removed, leaving 25 616 records from nine countries, across 39 sites, for 58 devices and 180 end-users. The characteristics of the participating sites are shown in Table 1. Most tests were done in fixed clinics (13 892; 54.2%), followed by home-based testing (5913; 23.1%), mobile clinics (3924; 15.3%) and laboratories (1887; 7.4%). Tests were done by laboratory staff (8230; 32.0%), clinicians (8188; 32.0%) and lay workers (9198; 36.0%). Most tests were done on venous blood (15 596; 61.0%) and the remainder on capillary blood (10 020; 39.0%). The median number of users per device was 3 (interquartile range, IQR: 2–5); the median number of tests per device was 470 (IQR: 252–672) and the median number of tests per user was 106 (IQR: 50–216).

**Table 1:** Characteristics of participating sites, nine countries, 2011–2013

Country	Sample type	User type	Settings	Users, No.	Devices, No.	Tests, No.	Invalid tests, No. (%)
Central African Republic	Venous blood	Laboratory technician	1 clinic, 1 laboratory	10	3	1925	362 (18.8)
Democratic Republic of the Congo	Venous blood	Laboratory technician, clinician	2 laboratories, 2 clinics, 1 mobile clinic	25	5	1888	254 (13.4)
Guinea	Venous blood	Laboratory technician	2 clinics	10	2	1896	150 (7.9)
India	Venous blood	Laboratory technician	2 clinics	5	2	1235	60 (4.9)
Kenya	Venous blood	Laboratory technician, clinician	2 clinics, 2 laboratories, mobile clinics^a^	26	11	5717	670 (11.7)
Lesotho	Capillary blood	Clinician, lay worker	6 clinics, 3 mobile clinics	40	10	4434	681 (15.4)
Malawi	Venous blood	Clinician	Mobile clinics^a^	10	7	1520	358 (23.6)
Mozambique	Capillary blood	Lay worker	Mobile clinics	6	2	1554	136 (8.8)
South Africa	Venous, capillary	Clinician, lay worker	8 clinics, 3 mobile clinics	49	16	5447	683 (12.5)
**Total**	–	–	**39**	**180**	**58**	**25 616**	**3354 (13.1)**

^a^ Home-based testing.

### Invalid results

There were 3354 invalid results: (13.1%) overall; with 4.9% for India, 7.9% for Guinea, 8.8% for Mozambique, 11.7% for Kenya, 13.4% for the Democratic Republic of the Congo, 12.5% for South Africa, 15.4% for Lesotho, 18.8% for the Central African Republic and 23.6% for Malawi. There were 12.7% errors per device (IQR: 10.3–19.9) and 12.1% per user (IQR: 7.1–19.2).

### Source of errors

**Fig. 1:**
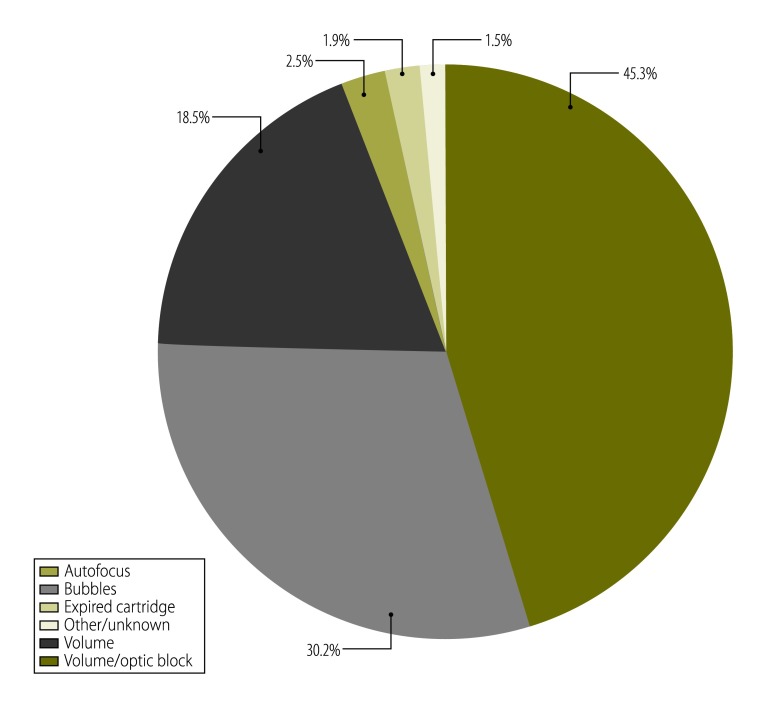
Types of error using the Alere Pima CD4+ analyser for CD4+ T-lymphocyte counts, (*n*=3354), nine countries, 2011–2013

**Fig. 2:**
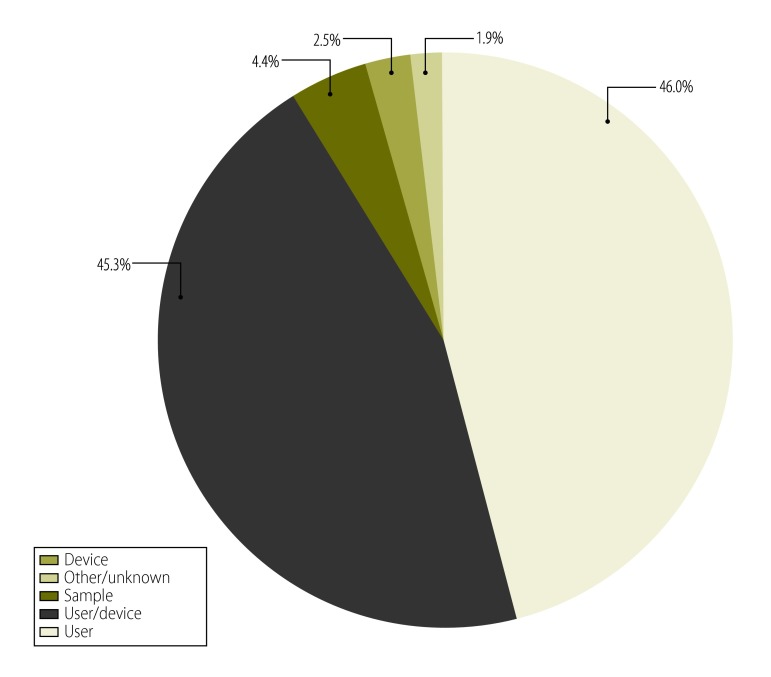
Sources of error using the Alere Pima CD4+ analyser for CD4+ T-lymphocyte counts, (*n*=3354), nine countries, 2011–2013

The most common errors were code 850 (37.0%) and code 880 (18.0%). These errors can result from multiple causes (Fig. 1). Based on the error codes, the source of the error was the user (1542; 46.0%); user or device (1519; 45.3%); device (83; 2.5%); or sample (147; 4.4%); with the remaining 63 errors (1.9%) being of unknown origin (Fig. 2). For calibration tests using Pima beads, 2% generated errors (207/10 404), with error codes 203 and 840 being the most common.

### Associated factors

The factors associated with invalid tests are shown in Table 2. Compared to South Africa, invalid tests were lowest in India (risk ratio; RR: 0.36; 95% confidence interval, CI: 0.27–0.46) and highest in Malawi (RR: 1.72; 95% CI: 1.50–1.97). Compared to tests done in 2013, there were fewer invalid tests in 2011 and 2012, but there was no significant secular trend. Users were significantly more likely to experience invalid results within their first 50 tests, but once they had done 50 tests or more, there was no trend towards a lower frequency of invalid results as their level of experience increased further. The frequency of errors was not correlated with the number of users per device.

**Table 2:** Factors associated with errors using the Alere Pima CD4+ analyser for CD4+ T-lymphocyte counts, nine countries, 2011–2013

Characteristic	RR (95% CI)
**Country**	
South Africa	(reference)
Central African Republic	1.47 (12.9–1.66)
Democratic Republic of the Congo	1.04 (0.90–1.20)
Guinea	0.60 (0.50–0.71)
India	0.36 (0.27–0.46)
Kenya	1.02 (0.92–1.13)
Lesotho	1.19 (1.07–1.32)
Malawi	1.72 (1.50–1.97)
Mozambique	0.65 (0.54–0.78)
**Time period**	
2013	(reference)
2011	0.88 (0.77–1.02)
2012	0.81 (0.74–0.89)
**Cumulative operator experience**	
1–49 tests	(reference)
50–99 tests	0.86 (0.78–0.94)
100–199 tests	0.88 (0.81–0.97)
≥ 200 tests	0.90 (0.83–0.99)
**Operators per device**	
1 operator	(reference)
2–3 operators	1.06 (0.97–1.17)
4–5 operators	1.09 (0.98–1.20)
≥ 6 operators	0.93 (0.83–1.03)
**Sample Type**^a^	
Venous blood	(reference)
Capillary blood	0.46 (0.40–0.53)

CI: confidence interval; RR: risk ratio.

^a^ Sub-analysis restricted to South Africa.

In clinics in South Africa, errors were significantly less frequent for lay health workers than clinicians (9.9% versus 19.8%; *P* = 0.001). Among laboratory technicians, the association between setting and the frequency of invalid tests differed by country. In the Central African Republic, invalid tests among laboratory technicians were more frequent at the clinic than at the laboratory (22.4% versus 16.3%; *P* < 0.001), while in the Democratic Republic of the Congo, invalid tests among laboratory technicians were less frequent at the clinic than at the laboratory (13.2% versus 18.7%; *P* < 0.001). Among lay health workers in Lesotho, Mozambique and South Africa, the association between setting and the frequency of invalid tests differed by country: in South Africa and Lesotho, the frequency of invalid tests was comparable in mobile clinic and fixed clinic settings (9.0% versus 9.9% in South Africa; *P* = 0.35; and 15.5% versus 16.1% in Lesotho; *P* = 0.66). However, in Mozambique, the frequency of invalid tests in mobile clinics was considerably lower than in fixed clinics (4.1% versus 13.6%; *P* < 0.001).

### Blood sample type

There were fewer invalid results when capillary blood samples were used (12.0%, versus 14.0% for venous blood samples; *P* < 0.001). In a subgroup multivariate analysis restricted to South Africa, (the only country in which both sample types were used) this association was strengthened (RR: 0.46; 95% CI: 0.40–0.53).

## Discussion

Under routine field conditions, the proportion of invalid CD4+ test results ranged from 5% in India to 24% in Malawi. Previous studies have reported comparable ratios from 5% in Thailand to 19% in South Africa.^15^^,^^33^ In our study, invalid results were slightly more frequent in venous blood samples than capillary blood samples. The use of a pipette to fill the device cartridge may generate air bubbles; since the CD4+ test is based on image detection, the presence of air bubbles may affect the results. To decrease the generation of air bubbles, the manufacturer recommends that capillary tubes instead of pipettes be used to transfer venous blood to the device. Studies in Senegal and Uganda reported more errors using capillary blood specimens (14% and 18%) compared to venous blood specimens (5% and 8%).^31^^,^^44^ However, other studies using venous blood have also found high ratios of invalid results: 10% in Ethiopia, 11% in USA and 15% in South Africa.^16^^,^^18^^,^^45^ Some studies have shown an increased variability of Alere Pima CD4+ results when capillary blood is used.^20^^,^^44^^,^^45^ However, a recent systematic review and meta-analysis found that capillary blood yielded more accurate results than venous blood.^47^ The fact that errors were more likely to occur using venous blood in our study may be related to the pipetting skills of laboratory staff. Therefore, training of laboratory staff in pipetting skills is as important as training clinic staff to perform capillary bleeds required for field testing.

The proportion of invalid samples for calibration tests was low (2%); however, in some instances, operators did not know how to interpret the results, so that testing continued despite clear indication of a problem; this calls for retraining. Multiple factors probably contribute to the wide variability of invalid results among countries, sites, devices and operators in our study. We did not find a clear association between the number of operators per device, the type of operator or the setting. Prospective studies under field conditions, specifically designed to evaluate the effect of various factors on the invalid tests are needed.

There were several limitations resulting from the retrospective nature of our study. We could not analyse errors according to cartridge lot number because this information was not contained in the cartridge barcode and sites did not document lot numbers systematically. It proved impossible to identify lot numbers via orders, as they were placed through various procurement centres in Europe as well as purchased from local distributors. We were unable to link results of repeat testing due to errors, so it was not possible to estimate the proportion of valid or invalid results after a second test. During the study period, none of the sites made use of online data systems for the analyser. Remote analysers can be connected to a centralised data warehouse via the internet, which could improve the timeliness of monitoring.

The high proportion of invalid CD4+ test results, necessitating the use of a second cartridge, raises the cost of testing per patient.^49^ Overall, 3354 tests had to be repeated; at a current price of 6 United States dollars (US$) per test, this translates to a cost of US$ 20 124. Repeat tests take time to process, decrease throughput, and oblige the patient to provide another blood sample if capillary sampling is used.

Training, monitoring, quality assessment and troubleshooting are essential aspects of point-of-care testing.^50^ It is important that post-market surveillance is done regularly and manufacturers continue to make the necessary improvements to their technologies and to supply service, maintenance and training for the full lifespan of the device.

In conclusion, 13.1% of results from the Alere Pima CD4+ analyser were invalid in this study. Most errors could be attributed to the operator but elucidating the exact cause proved to be difficult. Analyser errors are frequent and need to be factored into the implementation and operational cost for routine CD4+ testing.
